# Cardiac Magnetic Resonance Findings and NT-proBNP in Heart Failure with Reduced Ejection Fraction

**DOI:** 10.3390/tomography12090128

**Published:** 2026-09-08

**Authors:** Kemal Bugra Memis, İffet Doğan, Defne Şahin, Ali Osman Gulmez, Ece Özer, Mecit Kantarci

**Affiliations:** 1 Department of Radiology, Faculty of Medicine, Erzincan Binali Yıldırım University, Erzincan 24100, Türkiye; kemalbugramemis@gmail.com (K.B.M.); aliosmangulmez.2@gmail.com (A.O.G.); 2 Department of Radiology, Istanbul Mehmet Akif Ersoy Thoracic and Cardiovascular Surgery Training and Research Hospital, University of Health Sciences, Istanbul 34303, Türkiye; iffetdogan@gmail.com; 3 Department of Diagnostic and Interventional Radiology, Faculty of Medicine, Bezmialem Vakif University, Istanbul 34093, Türkiye; defnesumsahin@gmail.com; 4 Department of Radiology, Şişli Etfal Research and Training Hospital, Istanbul 34371, Türkiye; ece.ozer@saglik.gov.tr; 5 Department of Radiology, Faculty of Medicine, Atatürk University, Erzurum 25240, Türkiye

**Keywords:** HFrEF, T2 signal intensity ratio, extracellular volume, late gadolinium enhancement, left atrial diameter, myocardial remodeling

## Abstract

**Background/Objectives**: N-terminal pro-B-type natriuretic peptide (NT-proBNP) is an established heart failure biomarker, but its associations with cardiac magnetic resonance (CMR) parameters in patients with reduced ejection fraction remain unclear. This study examined the associations between serum NT-proBNP concentrations and CMR-derived functional, structural, and tissue characterization parameters in patients with HFrEF and evaluated which imaging parameters remained associated with NT-proBNP after multivariable adjustment. **Methods**: This retrospective single-center study included 92 consecutive patients with HFrEF who underwent CMR and NT-proBNP measurement within a 24 h window, with a median absolute interval of 6 h (range, 1–15 h) between the two assessments. CMR analysis included left ventricular ejection fraction (LVEF), left ventricular and left atrial (LA) cavity diameters, myocardial mass, extracellular volume fraction (ECV), the late gadolinium enhancement (LGE) burden, and the T2 signal intensity (T2-SI) ratio. Spearman correlation, univariate linear regression, and multivariable linear regression analyses were performed using log-transformed NT-proBNP values. **Results**: Log NT-proBNP demonstrated significant correlations with LVEF (r_s_ = −0.359, *p* < 0.001), T2-SI ratio (r_s_ = −0.332, *p* = 0.001), ECV (r_s_ = 0.383, *p* < 0.001), and LA diameter (r_s_ = 0.347, *p* < 0.001). No statistically significant correlations were observed between log-transformed NT-proBNP and LGE burden, LV cavity diameter, myocardial mass, LV wall thickness, age, or the recorded comorbidities. In multivariable analysis, LVEF, T2-SI ratio, and LA diameter remained associated with NT-proBNP after adjustment for the available covariates. **Conclusions**: Lower LVEF, a lower T2-SI ratio, and a larger LA diameter were associated with higher NT-proBNP levels in patients with HFrEF. The T2-SI ratio and LA diameter should be considered exploratory imaging markers requiring prospective validation.

## Simple Summary

In this retrospective, single-center study of 92 patients with heart failure and reduced ejection fraction, we examined the associations between cardiac magnetic resonance findings and NT-proBNP levels. A lower LVEF, a lower T2-SI ratio, and a larger LA diameter remained associated with higher NT-proBNP levels after adjustment for the available clinical and imaging covariates. These cross-sectional findings do not establish causality or prognostic value. The T2-SI ratio and LA diameter should be considered exploratory imaging markers that require validation in larger prospective multicenter studies.

## 1. Introduction

Heart failure with reduced ejection fraction (HFrEF) continues to be a predominant cause of morbidity and mortality, despite improvements in current medical treatments [1]. Precise evaluation of disease severity and myocardial remodeling is essential for risk stratification and personalized treatment [1,2]. Serum biomarkers and cardiac imaging methods yield significant prognostic findings, reflecting distinct facets of the disease process and are normally evaluated separately [3,4,5].

N-terminal pro-B-type natriuretic peptide (NT-proBNP) is a recognized biomarker for myocardial wall stress, commonly utilized in the diagnosis, prognostic evaluation, and treatment monitoring of heart failure (HF). Nonetheless, NT-proBNP levels are affected by various clinical factors and do not directly reflect the underlying myocardial substrate [3,5,6]. Cardiac magnetic resonance (CMR) imaging offers thorough tissue characterization alongside an accurate evaluation of ventricular function and morphology. Quantitative CMR parameters, such as left ventricular ejection fraction (LVEF), extracellular volume fraction (ECV), late gadolinium enhancement (LGE), and T2-based indices, yield critical insights into myocardial fibrosis, edema, and structural remodeling [7,8].

Previous studies have established that circulating NT-proBNP concentrations are associated with conventional indicators of heart failure severity, including reduced LVEF and adverse ventricular remodeling. CMR studies have also demonstrated associations between NT-proBNP and markers of myocardial fibrosis, including ECV and LGE, although the strength and independence of these relationships have varied across different populations and analytical models [9,10,11]. Left atrial enlargement, as a marker of chronically elevated filling pressure, has likewise been associated with increased natriuretic peptide concentrations [11]. Therefore, associations of NT-proBNP with systolic dysfunction, myocardial fibrosis, and atrial remodeling have already been described.

In contrast, evidence regarding the T2 signal intensity (T2-SI) ratio in chronic HFrEF remains limited. This semi-quantitative parameter was originally developed for the assessment of myocardial inflammation and reflects myocardial signal intensity relative to skeletal muscle [11,12]. Its relationship with NT-proBNP and whether this association persists after adjustment for conventional CMR parameters have not been sufficiently characterized in patients with chronic HFrEF. Furthermore, it remains unclear whether the T2-SI ratio and LA diameter retain associations with NT-proBNP when simultaneously evaluated alongside LVEF, ECV, LGE burden, and other structural CMR measurements.

Accordingly, this study aimed to examine the associations between serum NT-proBNP levels and CMR-derived functional, structural, and tissue characterization parameters in patients with HFrEF, with particular emphasis on the T2-SI ratio and LA diameter.

## 2. Materials and Methods

### 2.1. Ethical Approval

The study protocol was approved by the local Institutional Review Board (Clinical Research Ethics Committee of Erzincan Binali Yıldırım University, protocol number: 2026-12/12, date: 25 June 2026). Written informed consent for the anonymous use of CMR images and relevant clinical data for research purposes was obtained from all participants.

### 2.2. Study Population

This retrospective observational study included consecutive adult patients with HFrEF who underwent CMR imaging for various clinical indications between 1 June 2024 and 1 June 2026, as identified from the institutional database. The inclusion criteria were age ≥ 18 years, a diagnosis of HFrEF, and an NT-proBNP measurement obtained within 24 h before or after the CMR examination. Patients without an NT-proBNP measurement within this predefined 24 h window or with non-diagnostic CMR examinations due to substantial imaging artifacts or missing required sequences were excluded. Furthermore, all patients included in the analysis were evaluated as outpatients.

HFrEF was defined as an LVEF ≤ 40%, in accordance with current guidelines [6].

### 2.3. CMR Protocol

All CMR examinations were performed using a standardized protocol on a 1.5-T MRI scanner (Magnetom Aera, Siemens Healthineers, Erlangen, Germany) with an 18-channel cardiac coil. Long-axis and contiguous short-axis cine balanced steady-state free precession (bSSFP) images were acquired for the assessment of ventricular function, myocardial mass, wall thickness, and left atrial (LA) and left ventricular (LV) chamber dimensions.

For ECV calculation, native and post-contrast T1 maps were acquired in a single mid-ventricular short-axis slice using modified Look-Locker inversion recovery (MOLLI) sequences. Native T1 mapping was performed using a 5(3)3 acquisition scheme, whereas post-contrast mapping was performed approximately 10 min after contrast administration using a 4(1)3(1)2 scheme.

A gadolinium-based contrast agent was administered intravenously at a dose of 0.1 mmol/kg. LGE images were acquired approximately 15 min after contrast administration using a phase-sensitive inversion recovery (PSIR) sequence (TR/TE, 9/3 ms; flip angle, 25°; voxel size, 1.3 × 1.3 × 6 mm^3^). The inversion time was individually adjusted within a range of 250–350 ms to null the signal of normal myocardium.

For T2-SI ratio assessment, short-axis T2-weighted black-blood turbo inversion recovery magnitude (TIRM) images were acquired using the following principal parameters: TR/TE, 680/69 ms; inversion time, 170 ms; field of view, 360 × 275 mm; matrix, 125 × 272; voxel size, 1.3 × 1.3 × 6 mm^3^; slice thickness, 6 mm; and turbo factor, 20.

### 2.4. Image Analysis

CMR images were analyzed using Argus software (Siemens Healthcare, Erlangen, Germany) by radiologists who were blinded to the clinical and laboratory findings. Measurements were initially performed by a radiologist with 10 years of cardiac imaging experience and reviewed by a senior radiologist with 14 years of experience. Disagreements were resolved by consensus with a third radiologist with 21 years of cardiac imaging experience. Independent measurements by two observers and repeated measurements by the same observer were not obtained for either the T2-SI ratio or LGE burden. Therefore, formal interobserver and intraobserver reproducibility analyses could not be performed for these parameters.

The diameter of the LA cavity, LVEF, LV wall thickness, LV cavity dimensions, LV volumes, and myocardial mass were measured using cine bSSFP images. The LV internal diameter was obtained at end-diastole in the mid-ventricular short-axis plane at the level of the papillary muscles by measuring the maximum internal cavity diameter perpendicular to the interventricular septum. The LA anteroposterior diameter was measured at ventricular end-systole, when the left atrium reaches its maximum dimension, as the maximal distance from the posterior atrial wall to the midpoint of the mitral annular plane. The LV wall thickness was assessed using end-diastolic mid-ventricular short-axis cine images. The end-diastolic thickness of the interventricular septum was measured, excluding papillary muscles and trabeculations. LV contours were traced from cine CMR images, excluding the papillary muscles, to determine end-diastolic and end-systolic volumes (EDV and ESV), left ventricular ejection fraction (LVEF), and myocardial volume. Myocardial mass was calculated by multiplying myocardial volume by a myocardial tissue density of 1.05 g/mL.

**Figure 1:**
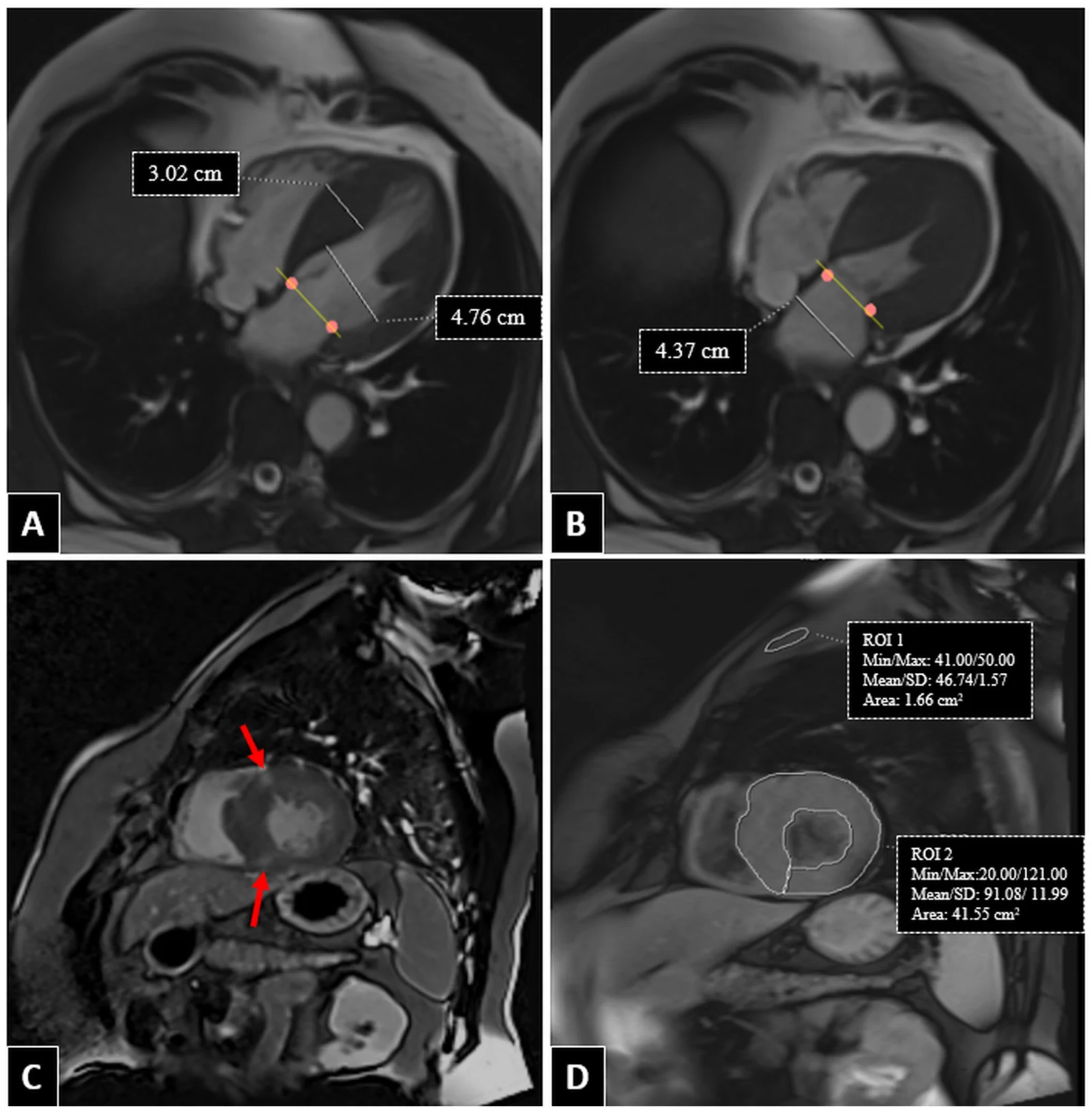
CMR images from a 40-year-old man with hypertrophic cardiomyopathy and LV systolic dysfunction (LVEF, 35%). (**A**) Four-chamber end-diastolic cine image showing marked interventricular septal thickening, measuring 3.02 cm (30.2 mm; short white line), and an LV cavity diameter of 4.76 cm (47.6 mm; long white line). The yellow line connecting the red dots indicates the level of the mitral valves. (**B**) Four-chamber end-systolic cine image showing an LA diameter of 4.37 cm (43.7 mm; white line). The yellow line connecting the red dots indicates the level of the mitral valves. (**C**) LGE image showing patchy nodular enhancement in the interventricular septum and at the right ventricular insertion points (red arrows). (**D**) Measurement of myocardial and serratus anterior muscle signal intensities on a T2-weighted TIRM image.

In inversion recovery analysis, the presence and extent of LGE were evaluated, and the LGE burden in the LV myocardium was determined. A segmental (semi-quantitative) method was used to determine the LGE burden. The left ventricle was divided into 17 segments, and the presence of LGE was assessed by examining three compartments (subendocardial, mid-wall, subepicardial) within each segment. The ratio of segments with positive LGE to the total segments was recorded as the LGE burden. Figure 1 shows measurements obtained from a patient included in the study group and diagnosed with hypertrophic cardiomyopathy.

**Figure 2:**
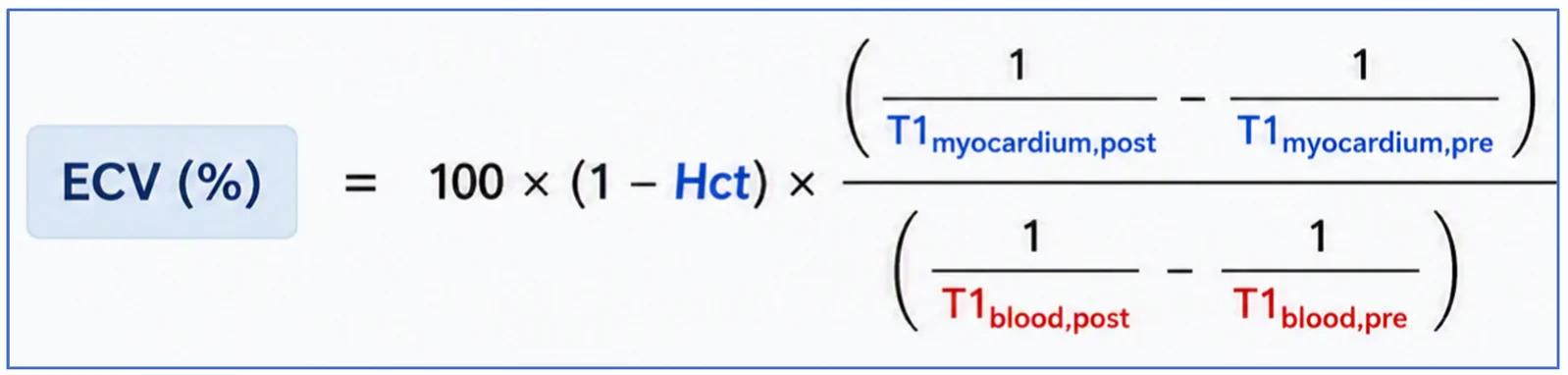
Illustration of the calculation of extracellular volume fraction (ECV) based on native and post-contrast T1 relaxation times of the myocardium and blood pool, alongside hematocrit levels.

ECV was calculated from native and post-contrast T1 maps acquired at the midventricular short-axis level. Myocardial and blood-pool ROIs were placed on the corresponding maps, with care taken to avoid partial-volume effects and contamination from adjacent tissues. ECV was calculated using the contemporaneous hematocrit value and the formula shown in Figure 2.

The T2-SI ratio was measured on three consecutive mid-ventricular short-axis T2-weighted black-blood TIRM images. On each slice, a freehand ROI was placed within the analyzable LV myocardium. A fixed ROI size or predefined myocardial segment was not used; instead, the ROI was adapted to the available myocardial area on each image. The ROI boundaries were maintained within the endocardial and epicardial contours to avoid contamination from the blood pool, epicardial fat, and partial-volume effects. Areas affected by visible motion, susceptibility, or coil-related signal inhomogeneity were excluded. On the corresponding slices, a separate freehand ROI was drawn within the homogeneous portion of the serratus anterior muscle. The ROI included as much visible muscle tissue as possible while avoiding the peripheral fascia, intermuscular fat, vessels, and areas of signal artifact. The myocardial and skeletal muscle signal intensities obtained from the three slices were averaged separately, and the T2-SI ratio was calculated as mean LV myocardial signal intensity divided by mean serratus anterior muscle signal intensity. This approach was based on the signal intensity ratio method described in the original 2009 Lake Louise Criteria [12].

### 2.5. NT-proBNP Measurement

An independent investigator, who did not participate in the CMR image analysis and was blinded to all imaging findings, extracted serum NT-proBNP measurements obtained within 24 h before or after the CMR examination from the electronic medical records. The actual interval between the two assessments was determined by comparing the electronic laboratory sampling timestamp with the recorded CMR acquisition start time and was expressed as the absolute time difference in hours. Demographic and clinical variables extracted from the electronic medical records included age, sex, underlying HFrEF etiology, hypertension, diabetes mellitus, and chronic kidney disease. Atrial fibrillation status and continuous eGFR values were not consistently available in the retrospective medical records and therefore could not be included in the analyses. HFrEF etiology was determined from the available clinical records and CMR reports.

### 2.6. Statistical Analysis

All statistical analyses were carried out using IBM SPSS Statistics (version 25.0; IBM Corp., Armonk, NY, USA). The normality of continuous variables was assessed using the Shapiro–Wilk test to guide the selection of appropriate statistical analyses. Continuous data were described as median with range (minimum–maximum) due to their non-normal distributions, whereas categorical variables were expressed as frequencies and proportions.

Because of its right-skewed distribution, NT-proBNP was transformed using the natural logarithm (ln) before correlation and linear regression analyses.

The relationship between log NT-proBNP and CMR-derived parameters was initially evaluated using Spearman’s rank correlation analysis. Variables demonstrating potential associations with log NT-proBNP were subsequently assessed by univariate linear regression analysis. Finally, multivariable linear regression analysis was performed to identify CMR parameters that remained associated with log NT-proBNP after adjustment for the remaining imaging variables. The multivariable linear regression model included age, sex, hypertension, diabetes mellitus, chronic kidney disease, LVEF, LGE burden, ECV, T2-SI ratio, LA and LV cavity diameters, LV wall thickness, and LV myocardial mass as potential clinical and imaging confounders. HFrEF etiology was not included in the model because the limited number of patients within individual etiological categories would have resulted in sparse subgroups and an unstable model. Multicollinearity among the candidate predictors was evaluated using tolerance and variance inflation factor (VIF) values. A tolerance value ≤ 0.20 or a VIF ≥ 5 was considered indicative of potentially important multicollinearity.

Unstandardized regression coefficients (B) with 95% confidence intervals (95% CIs), standardized regression coefficients (β), and corresponding *p* values were reported. A two-tailed *p* value of less than 0.05 was deemed statistically significant.

## 3. Results

**Figure 3:**
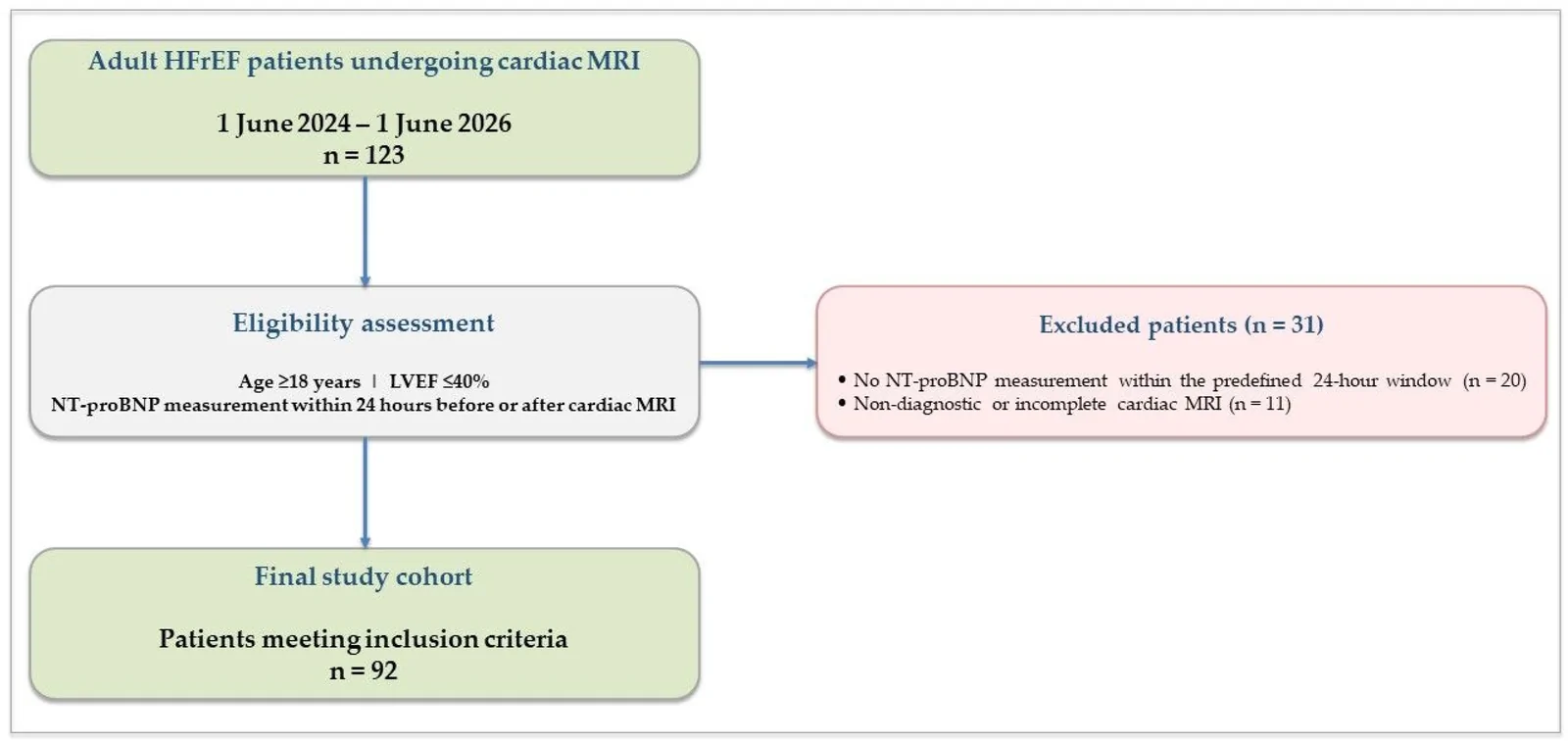
Flowchart of patient selection and study inclusion.

A total of 123 patients with HFrEF who underwent CMR between 1 June 2024 and 1 June 2026 were identified. Twenty patients were excluded because an NT-proBNP measurement within the predefined 24 h window before or after CMR was unavailable, and 11 were excluded because their CMR examinations were non-diagnostic owing to substantial artifacts or missing required sequences. The final study cohort comprised 92 patients (Figure 3). The median absolute interval between NT-proBNP sampling and the start of the CMR examination was 6 h (range, 1–15 h).

Of the 92 patients, 66 (71.7%) were male and 26 (28.3%) were female. The median age was 50 years, ranging from 22 to 80 years.

The underlying HFrEF etiologies were heterogeneous. Dilated and ischemic cardiomyopathies were the most frequent etiologies, each accounting for 28 patients (30.4%), followed by left ventricular noncompaction in 13 (14.1%), cardiac amyloidosis in 8 (8.7%), hypertrophic cardiomyopathy in 6 (6.5%), myocarditis in 4 (4.3%), and cardiac sarcoidosis in 3 (3.3%). Restrictive cardiomyopathy and unspecified heart failure were each recorded in one patient (1.1%). In the overall cohort, the median NT-proBNP concentration was 568 pg/mL (IQR, 273.8–1846.3 pg/mL; range, 31–11,805 pg/mL). Etiology-specific NT-proBNP distributions were presented descriptively in Table 1; no inferential comparisons were performed because several etiological subgroups contained few patients.

Only one patient (1.1%) had an NT-proBNP concentration below 125 pg/mL, with a value of 31 pg/mL, whereas the remaining 91 patients (98.9%) had NT-proBNP concentrations above this threshold. A logarithmic transformation was employed due to the right-skewed distribution of NT-proBNP levels (log NT-proBNP). NT-proBNP log values were analyzed in relation to LVEF, LV wall thickness, volumetric measurements, LGE data, and the diameters of the left atrial and ventricular cavities obtained from CMR imaging. The assessment outcomes are presented in Table 2.

One patient with ischemic cardiomyopathy had an ECV value of 80%. Reassessment of the original T1 maps, ROI placement, contemporaneous hematocrit value, and ECV calculation confirmed that this was not a transcription or calculation error. The predefined midventricular myocardial region used for ECV measurement contained extensive infarct-involved myocardium in this patient. The maximum ECV value among the remaining patients was 56%.

Log-transformed NT-proBNP was positively correlated with ECV and LA diameter and inversely correlated with LVEF and the T2-SI ratio. No statistically significant correlations were observed with age, sex, LV cavity diameter, LV wall thickness, LV myocardial volume, LV myocardial mass, or LGE burden.

The T2-SI ratio was inversely correlated with log-transformed NT-proBNP (rₛ = −0.332, *p* = 0.001). In the separate analysis of the ratio components, myocardial T2-TIRM signal intensity was inversely correlated with log-transformed NT-proBNP (rₛ = −0.330, *p* = 0.001), whereas serratus anterior muscle signal intensity was not (rₛ = −0.145, *p* = 0.167). Thus, the observed ratio association was unlikely to be explained solely by variation in the reference muscle signal.

In the univariable linear regression analyses, lower LVEF, lower T2-SI ratio, higher ECV, and greater LA diameter were significantly associated with higher log-transformed NT-proBNP levels. In the univariable linear regression analysis, each 0.1-unit increase in the T2-SI ratio was associated with a 0.305-unit decrease in log-transformed NT-proBNP (B = −0.305; standardized β = −0.423; R^2^ = 0.179; *p* < 0.001). After simultaneous adjustment for the evaluated CMR parameters, age, sex, hypertension, diabetes mellitus, and chronic kidney disease, LVEF (B = −0.044; 95% CI: −0.077 to −0.011; β= −0.295; *p* = 0.010), T2-SI ratio per 0.1-unit increase (B= −0.209; 95% CI: −0.346 to −0.071; β= −0.288; *p* = 0.003), and LA diameter (B = 0.045; 95% CI: 0.005–0.085; β= 0.212; *p* = 0.029) remained associated with log-transformed NT-proBNP. The association with ECV did not reach conventional statistical significance after multivariable adjustment (B = 0.037; 95% CI, −0.0001 to 0.075; standardized β = 0.242; *p* = 0.051). A comparison of the univariable and multivariable regression results for all evaluated CMR parameters is presented in Table 3. The multivariable model accounted for approximately 40% of the variance in log-transformed NT-proBNP (R^2^ = 0.395).

## 4. Discussion

In the present study, we evaluated the relationship between multiple CMR-derived structural and tissue characterization parameters and circulating NT-proBNP levels in patients with HFrEF. Several CMR parameters, including LVEF, ECV, LA diameter, and the T2-SI ratio, demonstrated significant correlations with NT-proBNP levels. However, after adjustment for the available clinical and imaging covariates, LVEF, T2-SI ratio, and LA diameter remained significantly associated with NT-proBNP.

The inverse relationship observed between LVEF and NT-proBNP is consistent with the established pathophysiology of HFrEF. Progressive deterioration of systolic function increases LV wall stress, resulting in enhanced myocardial secretion of natriuretic peptides. Numerous previous studies have demonstrated that lower LVEF is associated with higher circulating NT-proBNP concentrations and worse clinical outcomes [5,13,14]. In our cohort, lower LVEF was significantly associated with higher NT-proBNP levels in both the univariable and multivariable analyses, indicating that this relationship persisted after adjustment for the available clinical and imaging covariates.

Among the tissue characterization parameters, ECV showed a positive correlation with log-transformed NT-proBNP, consistent with previous studies linking diffuse interstitial expansion to increased myocardial stiffness and filling pressure [7,9,11,15]. However, this association did not reach statistical significance after multivariable adjustment.

LGE burden was not significantly associated with NT-proBNP in our study. Unlike ECV, which quantifies diffuse interstitial expansion, LGE primarily identifies focal replacement fibrosis. This difference may partly reflect the ability of ECV to characterize diffuse interstitial expansion, whereas LGE primarily identifies focal replacement fibrosis [16,17]. Moreover, the semi-quantitative assessment of LGE burden used in our study may have limited its sensitivity to detect subtle differences across patients.

The T2-SI ratio was inversely associated with log-transformed NT-proBNP after adjustment for the available covariates. This semiquantitative measure was originally introduced as part of the 2009 Lake Louise Criteria for assessing myocardial inflammation [12], but it has been less extensively studied in chronic HFrEF [18,19]. In the separate analysis of its components, myocardial T2-TIRM signal intensity was inversely correlated with log-transformed NT-proBNP, whereas serratus anterior muscle signal intensity was not. This finding makes it less likely that the ratio association was explained solely by variation in the reference muscle. Nevertheless, the biological basis of the inverse myocardial signal relationship remains unclear and should be investigated using quantitative T2 mapping in prospective cohorts.

LA diameter remained significantly associated with NT-proBNP after adjustment for the available clinical and imaging covariates. Chronic elevation of LV filling pressures results in progressive LA enlargement, making atrial size an integrated marker of long-standing diastolic burden [20,21]. Since NT-proBNP secretion is closely related to myocardial wall stress rather than isolated systolic dysfunction, the independent association observed in our study is biologically plausible. Previous studies have similarly demonstrated that left atrial enlargement is associated with increased natriuretic peptide concentrations and adverse clinical outcomes across different heart failure phenotypes [21,22].

CMR and NT-proBNP characterize different aspects of HFrEF. Recent advances in MRI acquisition and image analysis may improve the reproducibility and efficiency of quantitative CMR assessment [23]. The T2-SI ratio can be derived from routinely acquired T2-weighted images without contrast administration or additional image acquisition. However, its incremental clinical value cannot be determined from the present cross-sectional analysis and requires evaluation in prospective outcome studies.

Several limitations should be acknowledged. First of all, this was a retrospective single-center study with a relatively limited sample size, which may restrict the generalizability of the findings. The cross-sectional design precludes conclusions regarding causality or prognostic significance. Reliance on a single NT-proBNP measurement represents an additional limitation. Although all NT-proBNP measurements were obtained within 24 h before or after CMR and the median absolute interval was relatively short at 6 h, short-term biological variation in NT-proBNP levels during the interval between the two assessments cannot be entirely excluded. A major limitation was the absence of atrial fibrillation status and continuous eGFR data from the multivariable analysis. These variables could not be reliably obtained because they were not consistently documented in the retrospective records. Atrial fibrillation may independently increase circulating NT-proBNP concentrations and contribute to left atrial enlargement; therefore, the absence of atrial rhythm data may have materially confounded the observed association between LA diameter and NT-proBNP. Similarly, adjustment for chronic kidney disease as a binary variable does not adequately capture the continuous influence of renal function on NT-proBNP clearance and circulating concentrations. Accordingly, residual confounding by atrial rhythm and renal function cannot be excluded, and the adjusted associations, particularly that between LA diameter and NT-proBNP, should be interpreted cautiously and regarded as hypothesis-generating. NYHA functional class, body mass index, hemoglobin, volume status, and contemporaneous heart failure therapy were also not comprehensively available. Another major limitation was the absence of formal interobserver and intraobserver reproducibility analyses for both the T2-SI ratio and LGE burden. Although all measurements were performed by an experienced radiologist, reviewed by a senior radiologist, and resolved by consensus when necessary, independent measurements by two observers and repeated measurements by the same observer were not obtained. Consequently, interobserver and intraobserver reliability statistics could not be calculated. This is particularly relevant because T2-SI measurements may be affected by myocardial and skeletal-muscle ROI placement, artifact exclusion, and regional signal inhomogeneity, whereas the semi-quantitative assessment of LGE burden may be influenced by observer-dependent identification and segmental classification of enhancement. The heterogeneous etiologies of HFrEF represent another limitation, as the association between NT-proBNP and CMR parameters may differ across disease subtypes. Although HFrEF etiology was recorded, the limited number of patients within individual etiological categories precluded reliable etiology-specific analyses or adjustment for etiology in the multivariable model. The etiological heterogeneity of the study population should be considered when interpreting the observed associations. Tissue characterization parameters may vary according to the underlying myocardial substrate; for example, cardiac amyloidosis may be associated with marked extracellular expansion, whereas inflammatory cardiomyopathies may alter myocardial T2 signal. Therefore, part of the association between these parameters and NT-proBNP may reflect the etiological composition of the cohort. In addition, the relatively young median age and recruitment from a tertiary CMR center may have resulted in an overrepresentation of uncommon cardiomyopathies compared with an unselected community-based heart failure population. These factors may limit the generalizability of our findings. Ultimately, the T2-SI ratio is obtained from standard T2-weighted imaging instead of quantitative T2 mapping and should consequently be regarded as a semi-quantitative indicator. Images obtained from T2-weighted TIRM sequences exhibit heightened sensitivity to technical fluctuations and may be influenced by imaging parameters and the attributes of reference muscles.

## 5. Conclusions

In this cohort of patients with HFrEF, a lower LVEF, a lower T2-SI ratio, and a larger LA diameter were associated with higher NT-proBNP levels after adjustment for the available covariates. The T2-SI ratio and LA diameter may provide complementary information to conventional CMR parameters; however, their clinical relevance requires confirmation in larger prospective studies.

## Figures and Tables

**Table 1:** Distribution of NT-proBNP concentrations according to HFrEF etiology.

HFrEF Etiology	Patients *n* (%) *	NT-proBNP (pg/mL) M (Q1–Q3)	NT-proBNP (pg/mL) Min–Max Range
Dilated cardiomyopathy	28 (30.4)	553 (203–1184.5)	147–11,805
Ischemic cardiomyopathy	28 (30.4)	443 (208–1178.3)	140–10,378
Left ventricular noncompaction	13 (14.1)	2879 (561–4198)	249–7879
Cardiac amyloidosis	8 (8.7)	340.5 (257.8–1103.5)	154–1940
Hypertrophic cardiomyopathy	6 (6.5)	542.5 (305.5–926.5)	139–4772
Myocarditis	4 (4.3)	390.5 (225.3–1169.8)	31–3206
Cardiac sarcoidosis	3 (3.3)	1070 (1029–2197.5)	988–3325
Heart failure, unspecified	1 (1.1)	450	450
Restrictive cardiomyopathy	1 (1.1)	2478	2478
Overall	92	568 (273.8–1846.3)	31–11,805

* Percentages are based on the full cohort. M: median, Q1: 25th percentile, Q3: 75th percentile, Min–max: Minimum–maximum.

**Table 2:** Clinical and CMR characteristics of the study cohort and results of correlation analyses with log-transformed NT-proBNP.

		r_s_ Value	*p* Value *
**Patients, *n***	92		
**Sex** ***n* (%)**			
**Male**	66 (71.7)	0.041	0.699
**Female**	26 (28.3)		
**Age**			
Median (Min–Max)	50 (22–80)	0.152	0.147
**Comorbid conditions *n* (%)**			
**Hypertension**	38 (41.3)	0.145	0.085
**Diabetes mellitus**	10 (10.9)	0.099	0.150
**Chronic kidney disease**	7 (7.6)	0.020	0.395
**NT-proBNP Level (pg/mL)**			
Median (Min–Max)	568 (31–11,805)		
**Functional CMR parameters M (Min** **–** **Max)**			
LVEF	31 (18–40)	−0.359	<0.001
LV wall thickness (mm)	10 (6–30)	−0.015	0.887
LV cavity diameter (mm)	63 (43–92)	0.140	0.182
LV myocardial volume	77 (46–209)	0.084	0.427
LV myocardial mass (g)	81 (48–220)	0.088	0.407
LA cavity diameter (mm)	47 (30–63)	0.347	<0.001
**Tissue characterization metrics M (Min** **–** **Max)**			
LGE burden (%)	7 (0–18)	0.142	0.178
T2-SI ratio	1.40 (0.88–2.26)	−0.332	0.001
ECV (%)	33 (25–80)	0.383	<0.001

* Spearman rank correlations with log-transformed NT-proBNP. M: Median; Min–Max: Minimum- Maximum; r_s_: Spearman’s correlation coefficient; LV: Left ventricular; LA: Left atrial; EF: Ejection fraction; LGE: Late gadolinium enhancement; ECV: Extracellular volume fraction; CMR: Cardiac magnetic resonance.

**Table 3:** Univariable and multivariable linear regression analyses of factors associated with log-transformed NT-proBNP.

Variable	Univariable B (95% CI)	β	*p* Value	Multivariable B (95% CI)	β	*p* Value
**Clinical variables**						
Age, per 1 year	0.008 (−0.004 to 0.023)	0.015	0.270	0.005 (−0.011 to 0.029)	0.019	0.354
Female sex	0.031 (−0.012 to 0.042)	0.009	0.305	0.026 (−0.014 to 0.046)	0.068	0.296
Hypertension	−0.043 (−0.286 to 0.231)	0.031	0.402	−0.023 (−0.205 to 0.221)	−0.056	0.399
Diabetes mellitus	0.124 (−0.227 to 0.423)	0.048	0.854	0.098 (−0.145 to 0.153)	0.085	0.655
Chronic kidney disease	0.004 (−0.027 to 0.043)	0.025	0.258	0.002 (−0.014 to 0.033)	0.009	0.351
**Functional CMR parameters**						
LVEF, per 1%	−0.054 (−0.083 to −0.025)	−0.361	<0.001	−0.044 (−0.077 to −0.011)	−0.295	0.010
LV wall thickness, per 1 mm	−0.033 (−0.176 to 0.111)	−0.047	0.653	0.048 (−0.127 to 0.223)	0.070	0.587
LV cavity diameter, per 1 mm	0.033 (−0.001 to 0.067)	0.202	0.053	0.001 (−0.043 to 0.045)	0.006	0.966
LV myocardial mass, per 1 g	0.006 (−0.007 to 0.019)	0.094	0.370	−0.003 (−0.020 to 0.014)	−0.043	0.756
LA diameter, per 1 mm	0.072 (0.031 to 0.114)	0.343	<0.001	0.045 (0.005 to 0.085)	0.212	0.029
**Tissue characterization metrics**						
LGE burden, per 1 point	0.021 (−0.009 to 0.052)	0.144	0.170	−0.011 (−0.044 to 0.023)	−0.073	0.525
T2-SI ratio, per 0.1 unit	−0.306 (−0.444 to −0.169)	−0.423	<0.001	−0.209 (−0.346 to −0.071)	−0.288	0.003
ECV, per 1 percentage point	0.041 (0.010 to 0.072)	0.266	0.010	0.037 (−0.0001 to 0.075)	0.242	0.051

B: unstandardized regression coefficient; β: standardized regression coefficient; CI: confidence interval. The multivariable model simultaneously included all listed CMR parameters plus age, female sex (male reference), hypertension, diabetes mellitus, and chronic kidney disease. T2-SI coefficients are reported per 0.1-unit increase; other coefficients are per unit.

## Data Availability

The data are not publicly available because they contain potentially identifiable clinical and imaging information. De-identified data may be made available by the corresponding author upon reasonable request and subject to institutional and ethics approval.

## Abbreviations

The following abbreviations are used in this manuscript:

NT-proBNP	N-terminal pro–B-type natriuretic peptide
CMR	Cardiac magnetic resonance
LGE	Late gadolinium enhancement
ECV	Extracellular volume
HFrEF	Heart failure with reduced ejection fraction
